# Success Factors of Artificial Intelligence Implementation in Healthcare

**DOI:** 10.3389/fdgth.2021.594971

**Published:** 2021-06-16

**Authors:** Justus Wolff, Josch Pauling, Andreas Keck, Jan Baumbach

**Affiliations:** ^1^Chair of Experimental Bioinformatics, TUM School of Life Sciences, Technical University of Munich, Munich, Germany; ^2^Syte - Strategy Institute for Digital Health, Hamburg, Germany; ^3^LipiTUM, Chair of Experimental Bioinformatics, TUM School of Life Sciences, Technical University of Munich, Munich, Germany; ^4^Chair of Computational Systems Biology, University of Hamburg, Hamburg, Germany

**Keywords:** artificial intelligence, digital health, technology assessment, impact measurement, policy framework, success factor, public health

## Abstract

**Background:** Artificial Intelligence (AI) in healthcare has demonstrated high efficiency in academic research, while only few, and predominantly small, real-world AI applications exist in the preventive, diagnostic and therapeutic contexts. Our identification and analysis of success factors for the implementation of AI aims to close the gap between recent years' significant academic AI advancements and the comparably low level of practical application in healthcare.

**Methods:** A literature and real life cases analysis was conducted in Scopus and OpacPlus as well as the Google advanced search database. The according search queries have been defined based on success factor categories for AI implementation derived from a prior World Health Organization survey about barriers of adoption of Big Data within 125 countries. The eligible publications and real life cases were identified through a catalog of in- and exclusion criteria focused on concrete AI application cases. These were then analyzed to deduct and discuss success factors that facilitate or inhibit a broad-scale implementation of AI in healthcare.

**Results:** The analysis revealed three categories of success factors, namely (1) policy setting, (2) technological implementation, and (3) medical and economic impact measurement. For each of them a set of recommendations has been deducted: First, a risk adjusted policy frame is required that distinguishes between precautionary and permissionless principles, and differentiates among accountability, liability, and culpability. Second, a “privacy by design” centered technology infrastructure shall be applied that enables practical and legally compliant data access. Third, the medical and economic impact need to be quantified, e.g., through the measurement of quality-adjusted life years while applying the CHEERS and PRISMA reporting criteria.

**Conclusions:** Private and public institutions can already today leverage AI implementation based on the identified results and thus drive the translation from scientific development to real world application. Additional success factors could include trust-building measures, data categorization guidelines, and risk level assessments and as the success factors are interlinked, future research should elaborate on their optimal interaction to utilize the full potential of AI in real world application.

## Introduction

Artificial Intelligence (AI) is having the potential for a significant impact on the entire healthcare industry. Consequently, first governmental structures for Digital Health and subsequent AI scaling are currently being defined. For instance, the German government has published a national law for the reimbursement of registered Digital Health services by public health insurances (1, 2). Based on the growing amount of digital health applications, the high expectations related to medical, social, and economic improvements, as well as the need for digital health routines triggered by COVID-19, the success factors for AI implementation need to be defined now.

The academic literature elaborated in detail on the benefits and challenges of AI in healthcare. Already in 2015, Deo reported that “although there are thousands of papers applying machine learning algorithms to medical data, very few have contributed to clinical care” and potential obstacles for machine learning implementation require further research (3). In 2018, Park and Han provided methodological guidelines to evaluate the clinical performance of AI for medical diagnosis and prediction (4). In the same year, Yu et al. described different potential applications of AI and the clinical integration at different AI development stages (5).

In 2019, Triantafyllidis and Tsanas noted that still only few real world Digital Health intervention studies could be identified for their review of machine learning applications. However, the ones identified and analyzed were useful and effective (6). In the same year, Racine et al. highlighted substantial challenges concerning the use of AI, including dynamic information and consent, transparency and ownership, and privacy and discrimination (7). Furthermore, He et al. confirmed there are limited real-world AI applications, and the authors discussed various concrete and practical improvement areas related to data sharing, transparency of algorithms, data standardization and interoperability (8).

In 2020, Alhashmi et al. surveyed 53 health and IT specialists and highlighted the importance of managerial, organizational, operational and IT infrastructure related factors for AI applications (9).

Despite the substantial ongoing research regarding the benefits and improvement of AI in healthcare, there are only a few real-world application cases covered in academic research or openly published. These include, among others, major initiatives such as IBM's investment of over 4 billion USD into IBM Watson (10), and Amazon, which agreed with Cerner to establish a range of AI in healthcare services under Amazon Web Services (11). In addition, start-ups have also brought successful AI applications to the market. For example, the FDA approved deep learning platform Arterys or Babylon Health, which performs ~4,000 clinical consultations on their platform per day (8, 12).

From our perspective, a gap between the promising and comprehensive academic research on the high potential of AI in healthcare and the comparably low level of actual practical implementation can be observed. Despite previous recognition of this gap and isolated analyses of potential areas of improvement, this is the first attempt to systematically identify success factors that significantly facilitate the implementation of AI in healthcare based on previous academic research and real-world AI applications.

## Materials and Methods

First, the success factor categories and according database search queries have been defined and there are several success factors, that had already been researched in prior publications. For example in 2016, Ross et al. identified factors that influence the implementation of eHealth and found that the individual e-health technology, the outer setting, the inner setting, the individual health professionals as well as the process of implementation are key success factors (13).

**Figure 1:**
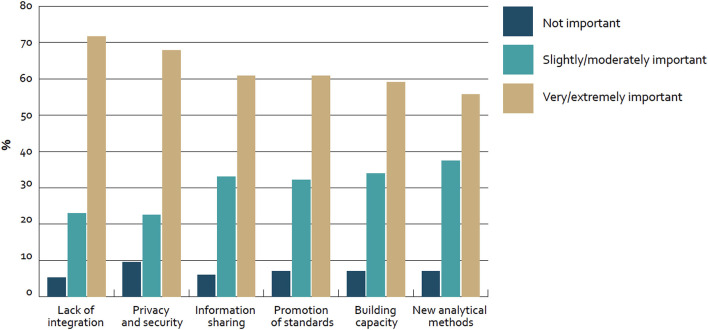
Barriers to adoption of big data for health globally—survey of 125 countries by the WHO (14).

In our case we derived the success factor categories from the Big Data section results of the “Global diffusion of eHealth: Making universal health achievable” report of the World Health Organization (WHO), as displayed in Figure 1. In this global survey with 125 WHO member countries the following results with regard to adoption barriers of Big Data were revealed (14).

Roughly 70% of countries mentioned “lack of integration” (72%; *n* = 81) and “privacy and security” (68%; *n* = 78) as very or extremely important barriers to adoption. Furthermore, about 60% of countries considered “information sharing” (61%; *n* = 70), “promotion of standards” (61%; *n* = 70), and “building capacity” (59%; *n* = 68) in the same category. In addition to that, “new analytical methods” were mentioned (55%; *n* = NA). Furthermore, only less than a fifth of all countries (17%; *n* = 21) reported to have a national policy or strategy regulating the use of big data in the health sector and thus from our perspective “Strategy setting” based on consequent impact measurement is also a key barrier for the adoption.

Based on these results three improvement categories have been deducted:

1) Technology (“Lack of integration,” “Privacy and security,” and “Information sharing”)
2) Policy (“Promotion of standards” and “Building capacity”)
3) Medical and economic impact (“New analytical methods” and “Strategy setting”)

Thus, in this paper success factors are defined as facilitators for AI implementation based on recommendations across the segments technology, policy as well as medical and economic impact.

### Academic Literature

Academic literature was accessed and identified via a research of the data base “Scopus” with the search terms “Artificial Intelligence,” “Healthcare,” “Health care,” “Success factor,” “Technology,” “Policy,” “Medical Impact,” and “Economic Impact” (Search term query: “artificial intelligence” AND “healthcare” OR “health care” AND “success factor” AND “technology” OR “policy” OR “medical impact” OR “economic impact”). Furthermore, since not every journal is included in Scopus and the defined success factor categories are covering a broad spectrum of journal types, additionally also the online public access catalog OPACplus of the Technical University Munich was used as second database.

The search term “Artificial Intelligence” has not been exchanged with other options like “Machine Learning” or “Neural Networks” as the term “Artificial Intelligence” has been used by far the most, according to the results of a Google Trend Analysis comparing the most frequently used search terms regarding AI in healthcare (15).

The following further inclusion and exclusion criteria were applied:

1) The research is published in a journal article.
2) The publication is written in the English or German language.
3) The publication date was between the years 2015 and 2020.

Further, in terms of content, they were included if at least one of the following content-related criteria were met:

1) Comprehensive description of an AI application.
2) Evaluation of the efficiency and outcomes of an AI application.
3) Description of a concrete real-world AI application.

Subsequently, publications were excluded from the analysis if they met any of the following criteria:

1) The title or abstract did not mention a topic related to AI.
2) The abstract did not contain a description of the AI application.
3) The full text did not elaborate on the implementation process of an AI application.

The query returned 1,494 hits out of which 1,081 were published between the years 2015 and 2020 in Scopus as well as 1,098 hits out of which 648 were published in the mentioned time frame in OpacPlus. Applying the listed in- and exclusion criteria, 26 publications qualified as a basis for the academic literature review in Scopus and 24 publications in OpacPlus.

### Real-World Cases

We identified real-world AI applications covered in academic literature using the abovementioned search approach. However, since only a small fraction of the practical AI implementation cases is covered by academic research, further real-world cases were identified through a Google-based advanced search for listings using the following search terms: “Artificial Intelligence,” “Healthcare,” and “Implementation.” Google listings were included if they fulfilled all of the following criteria:

1) The AI implementation description was uploaded within the last year (i.e., results between 1 April 2019 and 1 April 2020), and the described practical case was not implemented before 2015.
2) The AI implementation is written in English or German language.
3) The AI implementation has a clear identification of the real-word AI application (i.e., cited the name of the AI provider, the technology, and the implementation location or institution).

**Figure 2:**
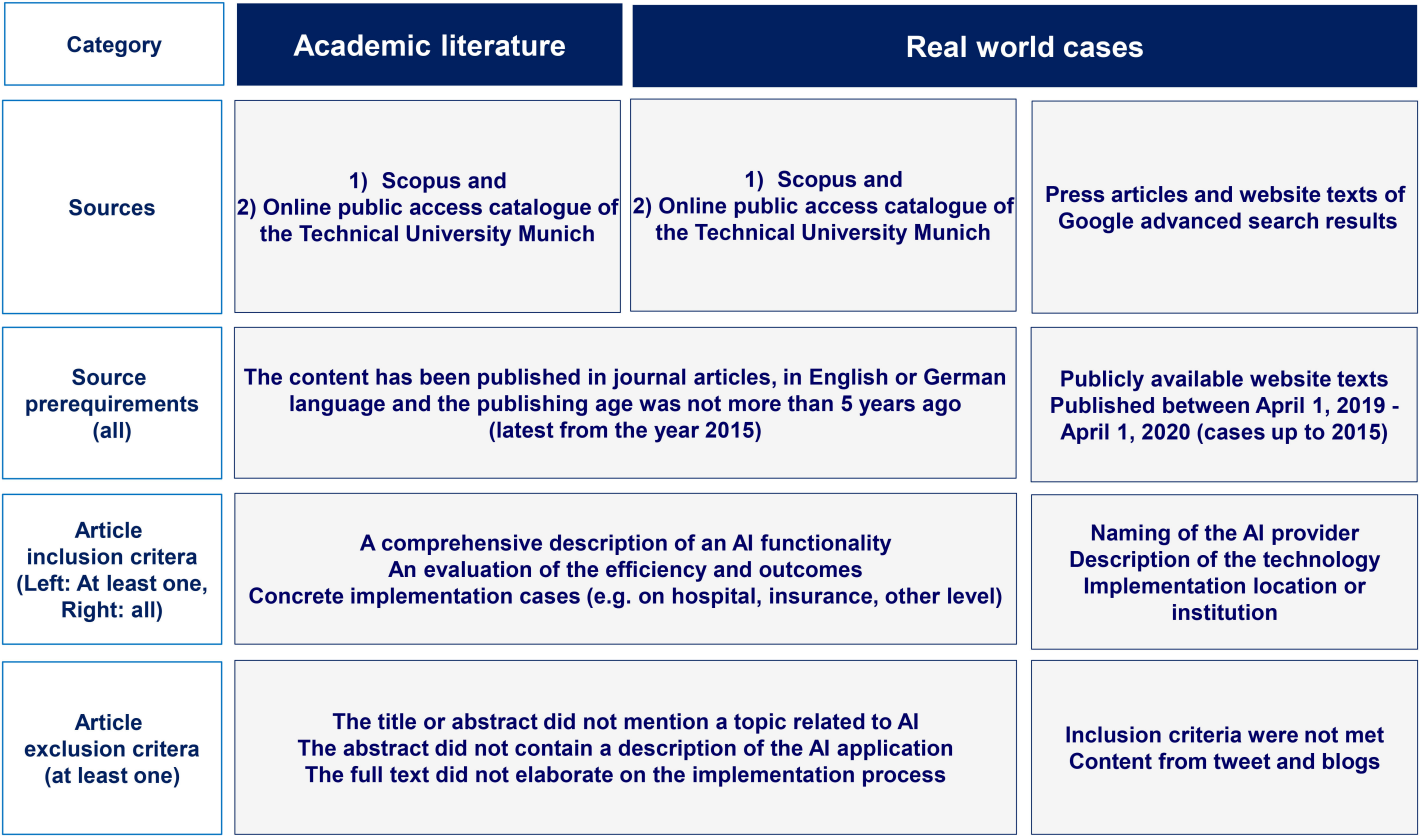
Methodology for the identification of academic literature and real-world AI application cases in healthcare (authors).

**Figure 3:**
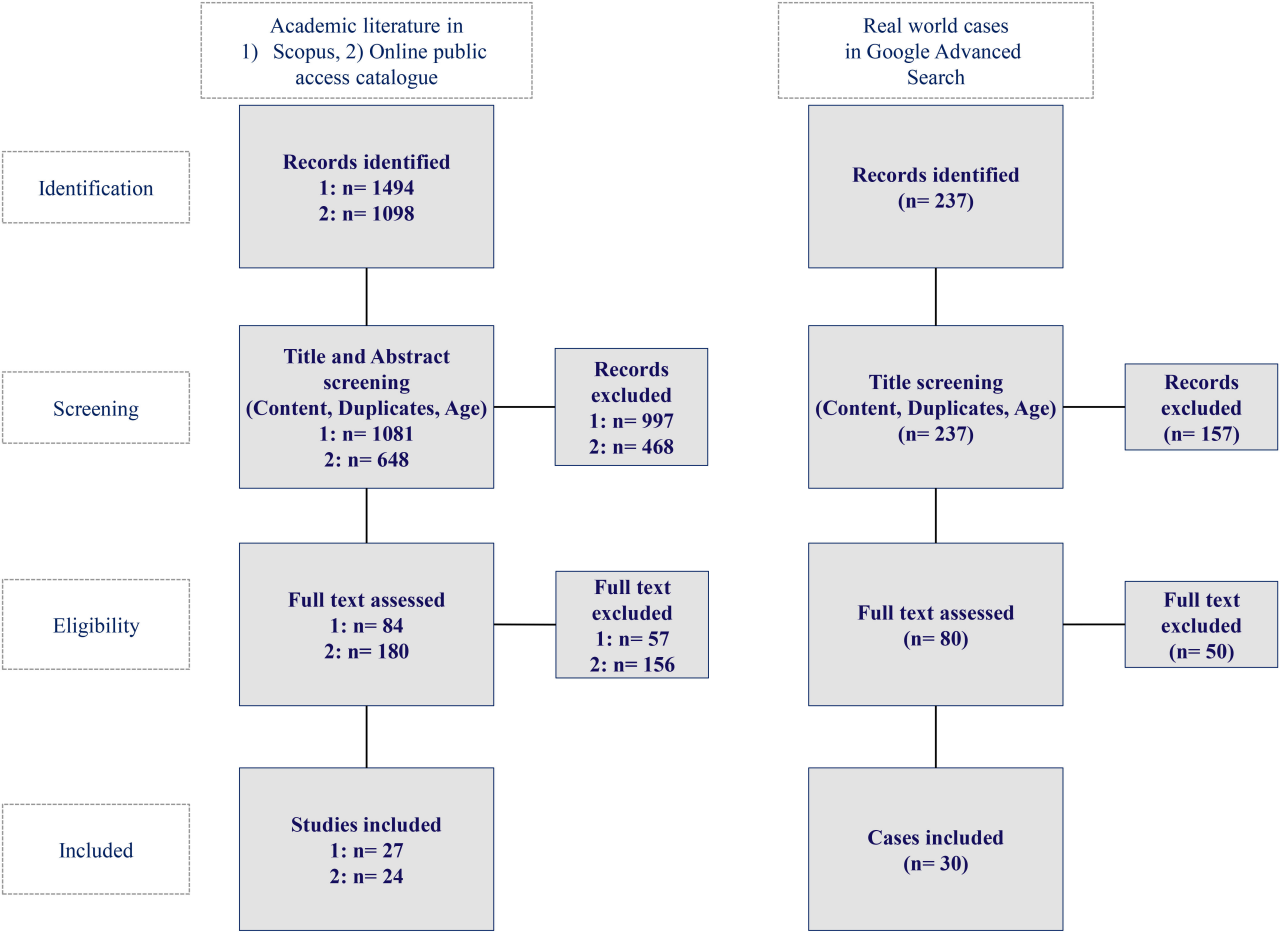
Prisma flow diagram for academic literature and real world case research (authors).

AI applications originating from tweets or blogs were excluded. The query yielded 237 hits in the Google advanced search, of which 30 hits qualified as a basis for our analysis of real-world AI applications in healthcare. Figure 2 depicts the methodology for the identification of academic literature and real-world AI application cases in healthcare while Figure 3 shows the Prisma flow diagram.

## Results

### Barriers to AI Implementation in Healthcare

**Figure 4:**
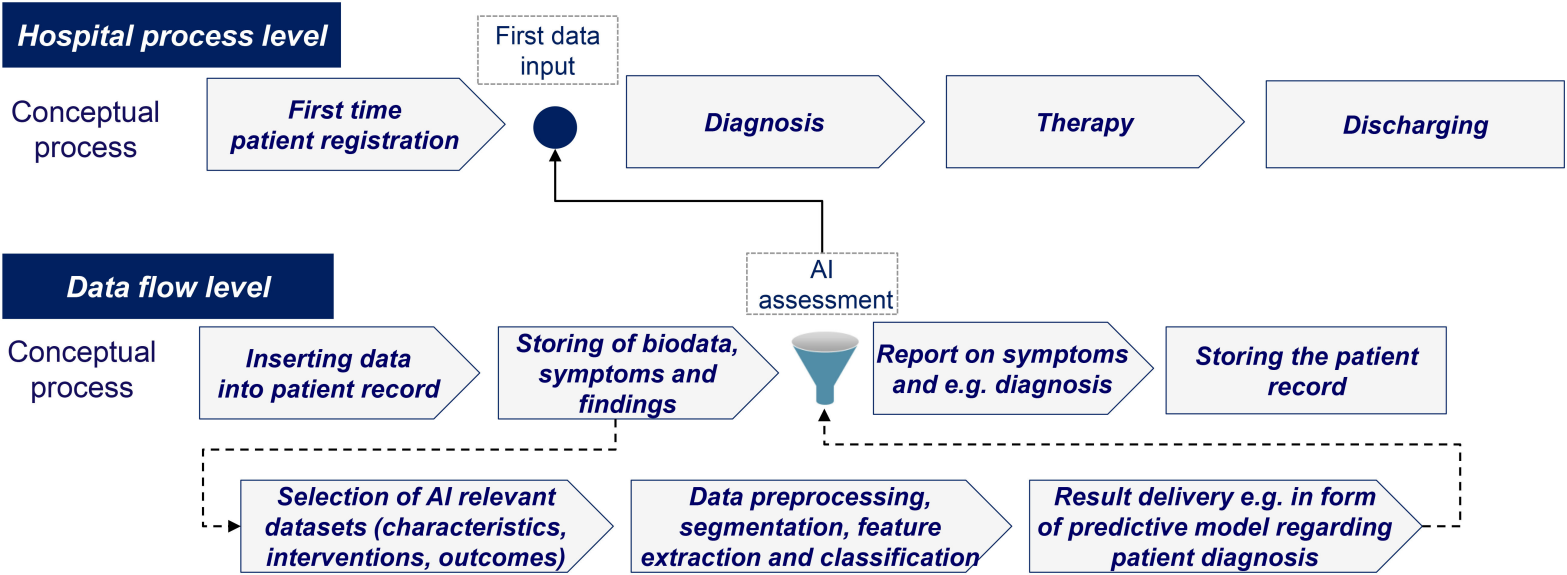
AI integration into routine healthcare processes (authors).

Based on the academic literature and real-world case analysis, various barriers to AI implementation were identified. Given the need to access large amounts of data under strict privacy regulations and the dependence on managerial acceptance, it became evident that AI implementation needs to be tailored further to fit into existing healthcare routines. An illustrative example of how AI can be integrated into routine healthcare processes is shown in Figure 4.

As described above, the key identified barriers for AI implementation relate to the following fundamental issues: (1) non-privacy focused technological implementation, (2) shortcomings in current policy settings, and (3) the lack of medical and economic impact measurements. As comparison, in a framework about the success factors for AI implementation in the telecommunication industry in China, the author concluded that three success factors apply, namely the external environment, e.g., government involvement or vendor partnerships, organizational capabilities, e.g., managerial or technical skills, and innovation attributes, e.g., compatibility or relative advantage (16).

Our first barrier consists of major technological limitations that constrain AI implementation. Notably, access to medical data is commonly too fragmented and limited to Electronic Health Record (EHR) data and the existing data silos in the healthcare provider context do not enable complete access for AI applications (17). Furthermore, some data material, though available and accessible, may not be useable because of a lack of precise data requirements. For instance, in medical image analysis, edges of pictures may be unclearly defined, or high noise may inhibit the analyses (5). Further examples show that AI for breast, lung and liver cancer detection would require significantly enhanced data preprocessing and image processing or that in general a much more facilitated integration into existing workflows of EHRs is required to foster the use of clinical decision support systems (18, 19).

The second barrier shows, that there are major policy deficiencies that inhibit AI implementation. In numerous countries, it is neither clear who the regulatory authority for AI in healthcare is, nor how the ever-changing black box of AI will be assessed from a policy perspective (13). The General Data Protection Regulation (GDPR) in the EU and the Food and Drug Administration (FDA) regulations in the US for general data handling are very specific. However, there are no overarching policies, reporting standards, or recommendations concerning AI in healthcare. It could even be argued that no specific regulatory authority would be needed, as for example there is also no dedicated authority for decision support systems or treatment algorithms. Still, due to the potential risks of applying black box AI algorithms, it can be expected that clinicians will request clear and comprehensive regulations for increased application.

The third barrier in form of the lack of clinical and economic impact measurement further contributes to the low level of practical implementation. Although performance metrics on the outcomes of AI, such as levels of accuracy of preventive care or recommendations for therapeutic decisions are abundant, medical and economic benefits are often not measured, or the measurement approach is not clearly defined (4). The strategy, business models and, especially, reimbursement as a core element for AI application in healthcare are thus, often still unclear (3).

### Success Factors for AI Implementation in Healthcare

#### Technological Implementation

The academic literature describes in detail the different technological categories of AI applications, ranging from natural language processing up to expert systems (20). In certain medical sectors, specific types of AI applications are more commonly applied, such as image analysis in radiology or dermatology (21). Most of the real-world AI application types face the challenge of combining practicality with privacy since they require complete data access.

This challenge could successfully be mitigated by several indication-focused practical cases of real-world AI applications. For instance, a “Persuasive Communication Tailoring” AI tool has been implemented to send motivational smoking cessation messages to adults. The machine learning version of the anti-smoking application significantly outperformed the prior rule-based system, and the algorithm was trained using data from messages, feedback databases, and user profiles (22). Another example is the pharmaceutical company MSD, which created an AI-driven communication channel based on the Facebook messenger for a chatbot about urgent matters in immune-oncology. The underlying conversational relationship between the physician and the chatbot is not bound to the data of EHRs, but is a stand-alone tool focused on the concrete problem-solution data access (23).

Furthermore, “privacy-by-design” technologies that aim to integrate privacy concepts in the design phase of an AI application, are increasingly being used (24). For example, at the institutional level, a health insurance system in Romania developed a GDPR compliant cloud-based AI application using a “SwarmESB-based” architecture with advanced data protection features. In the cloud infrastructure, multiple small entities are established, which possess one specific function for each task, such as ID copying, check of employment status, or retirement agency verification (25). Another reference case for privacy by design is “FeatureCloud,” a platform for the exchange of model parameters instead of raw data in a combined federated AI model (26). The technological implementation should consider the recommendations illustrated in Table 1.

**Table 1:** Comparison of a US and an EU framework related to AI (29, 30).

**Title**	**Proposed regulatory framework for modifications to Artificial Intelligence/Machine Learning (AI/ML)—Based Software as Medical Device (SaMD) Discussion paper and request for feedback (29)**	**Ethics guidelines for trustworthy AI (30)**
Key content (excerpts)	- Establishment of quality systems and Good Machine Learning Practices (GMLP), including usage of only relevant data, the separation between training, tuning and test datasets or transparency of the output - Conduction of initial pre-market reviews to assure safety and effectiveness - Monitoring of the AI devices based on development, validation, and execution of algorithm changes such as “Algorithm Change Protocol” - Post-market real-world evidence performance reporting for maximized safety and effectiveness	-Independent high-level expert group on artificial intelligence set up by the European Commission/April 8, 2019
		- Ethical principles as foundations of trustworthy AI (respect for human autonomy, prevention of harm, fairness, and explicability) - Seven key requirements of realizing reliable AI [(1) human agency and oversight, (2) technical robustness and safety, (3) privacy and data governance, (4) transparency, (5) diversity, non-discrimination, and fairness, (6) environmental and societal well-being and (7) accountability] - Assessing trustworthy AI (assessment list when developing, deploying or using AI systems)

##### Policy Setting

Previous publications cover a wide range of policy topics ranging from the dangers of so-called “black box” AI decisions to the paradigm shift from almost absolute protection of patient data to an economy of patient data sharing (27, 28). Nevertheless, there are almost no laws or standards that comprehensively regulate the use of AI in healthcare and there are significant geographical differences as shown in the US and EU propositions in Table 2.

**Table 2:** Key factors for AI technology development planning (authors).

**Application scenario differentiation**	**Data processing structure definition**	**Privacy by design and product class setting**
Indication-focused, e.g., smoking	Data access, e.g., EHR, wearables	AI technology implementation with a “privacy-by-design” structure
Institution-focused, e.g., health insurance	Data exchange pathways, e.g., connected vs. stand-alone	Compliance with medical product classification
Other	Data confidentiality measures, e.g., cloud infrastructure	Adaptability for changing AI regulatory requirements

The European Commission published also a risk-based legal adoption plan in the “White book for Artificial Intelligence” regarding training data, data storage, and human supervision (31).

In addition to the analysis of various regulatory frameworks, we also examined geographically independent policy factors.

First, it is expected, that AI, more generally, will evolve over several stages from the “Artificial Narrow Intelligence” to the “Artificial General Intelligence” up to “Artificial Super Intelligence,” and the according use cases will develop from stand-alone problem-solving over strategic decision-making up to independent strategy execution (32). To support this evolution of AI, one should differentiate between a permissionless approach, where innovation can be tested and problems are solved as they occur, and a precautionary approach, where AI applications are banned from the beginning if they impose a distinct risk (33). Therefore when defining policy principles, one can build on a “form follows function” (permissionless) and a “first frame then function” (precautionary) approach, where the permissionless approach is less restrictive for AI implementations.

Second, it should be taken into account that AI decision-making processes are different from human decision-making processes. AI is able to infer answers more quickly and accurately and to consider a significantly larger number of scenarios simultaneously, and can, thus, reach different decision outcomes. Furthermore, AI learns from “wrong” behavior, and the severity of such adverse experiences and failures varies from case to case. Consequently, AI decision outcomes can also differ from that of human (34). To assess the reasoning process, protocols are required for the status ante, the status quo concerning the time taken for a decision, the number of scenarios considered, and the accuracy of the result obtained by AI.

Subsequently, the responsibilities of different stakeholders in AI processes should be addressed. For instance, in the real-world case of AI-based automatic robotic surgery, it is required to differentiate between accountability, liability, and culpability (35). A clear task differentiation is necessary, so that accountability can be clearly defined based on the process steps (e.g., x-ray image analysis), liability can be limited (e.g., manufacturer, operator, maintenance) and culpability can be exclusively attributed (e.g., an obligatory second human check of a decision obtained by an AI application).

A practical case of a real-world AI application that follows a permissionless approach is the collaboration between Philips, Salesforce, and Radboud University Medical Center. In this context, the involved parties extracted specific medical datasets, such as cancer research or COPD, and established the cloud software “HealthSuite” as a database on which patients and physicians can store health data for authorized access (36, 37). The case complies with the regulatory requirements via data protection measures, and available data is currently used by ca. 40 deep learning researchers focusing on various topics like medical image analysis (38).

**Figure 5:**
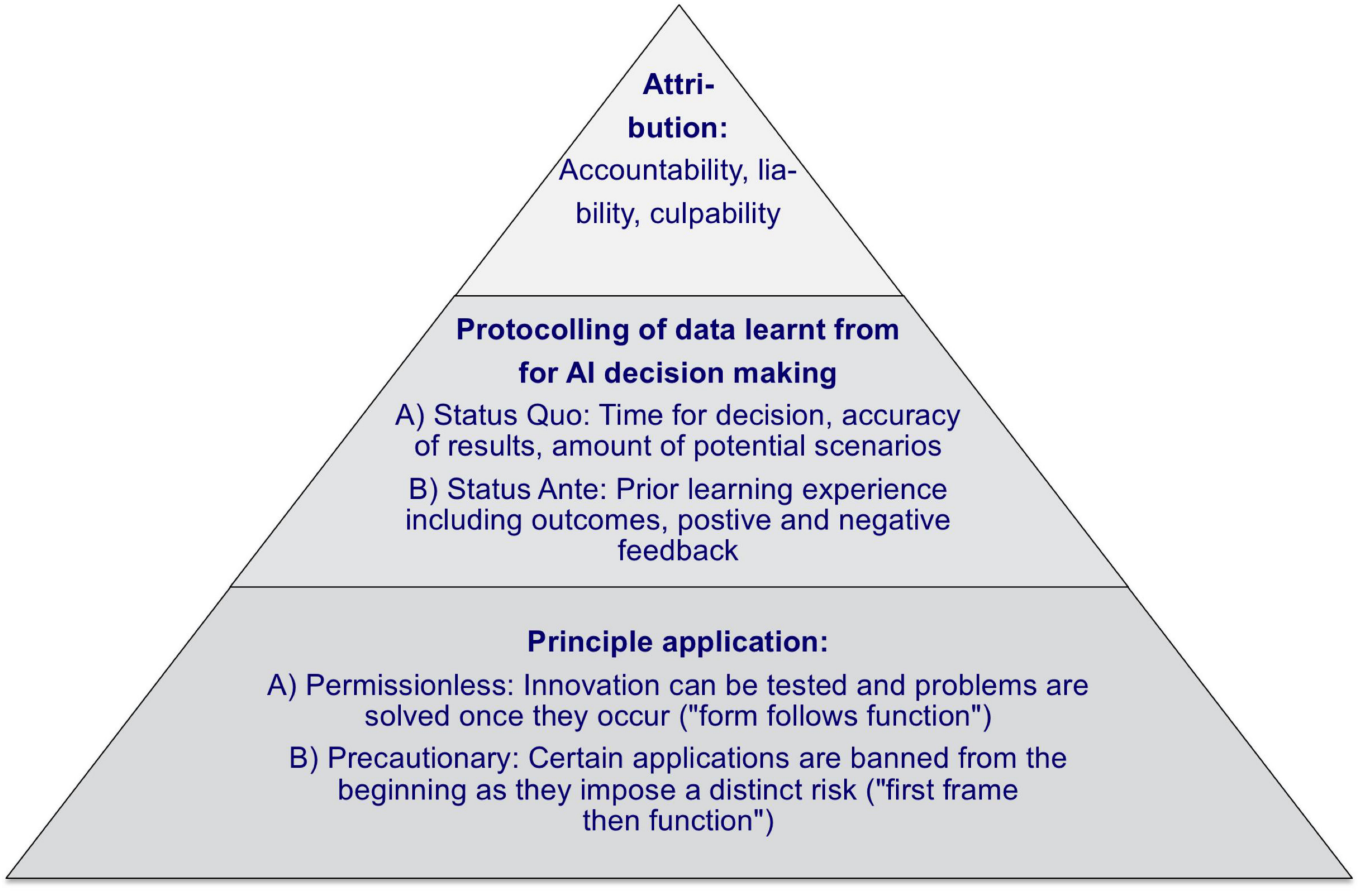
Policy framework pyramid (authors).

In an environment of continually evolving national and international recommendations that lack concrete implementation guidance, a comprehensive policy is needed. An overview about a potential policy framework structure is displayed in Figure 5.

##### Medical and Economic Impact Measurement

AI strategy setting and implementation is a decision that is based on medical and economic decisions. Previous research has demonstrated that there are generally too few economic impact evaluations and, that many available ones lack critical components such as a net present value calculation or a comparison of alternative AI applications (15). This is particularly relevant in light of the meaningful investment volumes in the area of AI in healthcare, especially by large corporate entities, and the difficult economic impact measurement led to the application of industry-specific evaluation methods (40). Consequently, precise, accurate and internationally applicable medical and economic impact measurements are required.

**Figure 6:**
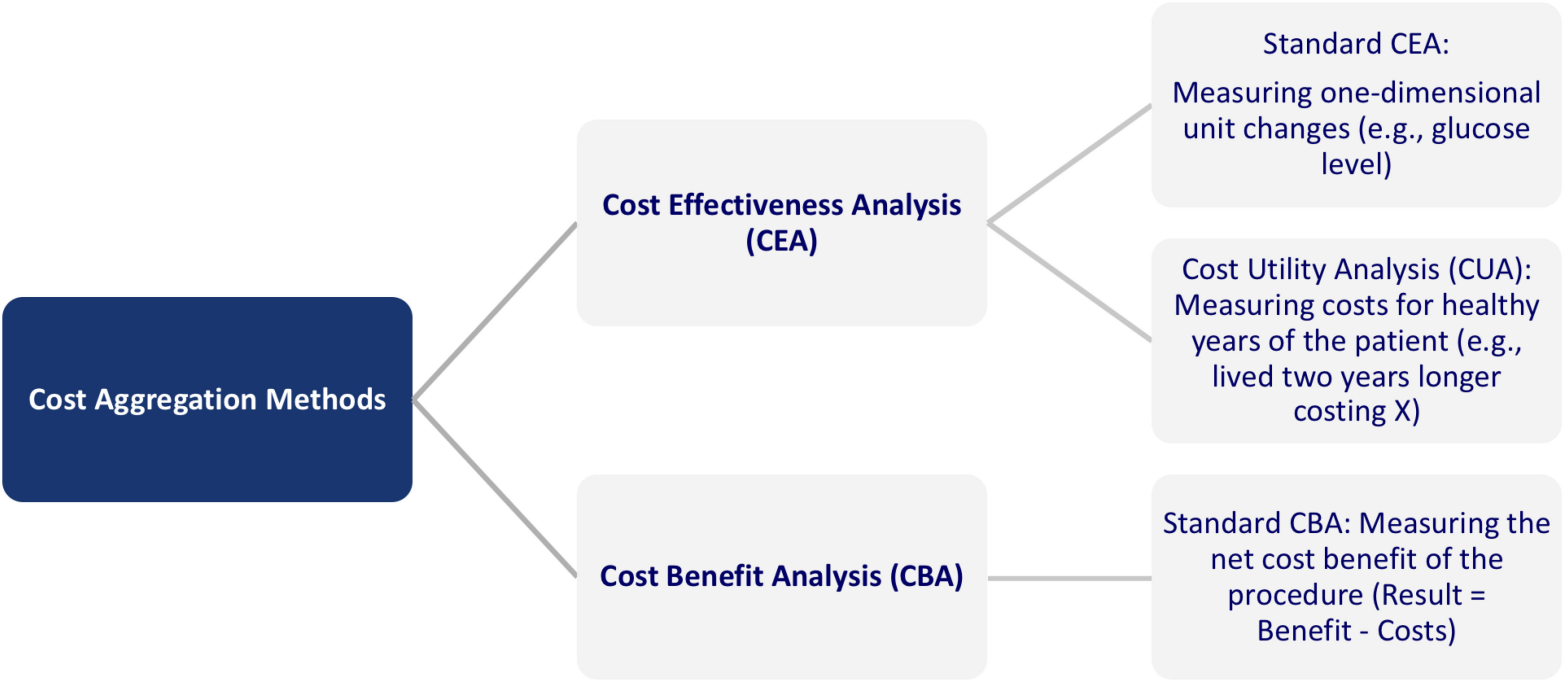
Overview of cost aggregation methods in healthcare (39).

The approaches to measure the outcomes of Digital Health, in general, and AI, in particular, can be classified into two categories: Cost Effectiveness Analysis (CEA) and Cost Benefit Analysis (CBA) (39). The first category can be further divided into standard CEA and Cost Utility Analysis (CUA). The CEA analysis refers to a cost comparison of a new vs. an old method, for example, regarding blood glucose measurement, wound size, or symptom-free days. In CUA, the outcome is measured in healthy years, for example, measured as quality-adjusted life years (QALYs). Specifically, QALYs provide an estimate of how many extra months or years of life, a person might gain by undergoing a specific treatment. Under a cost-minimization approach and the precondition of an equal medical outcome, different treatments can be compared. The difference between the approaches is that while the CBA can answer whether a new digital service is worthwhile, the CEA can answer the question of which of the alternative services is less costly to reach the equivalent outcome. Figure 6 provides an overview of the different categories.

For a large-scale implementation of AI in healthcare and to qualify for reimbursement on a broad scale across insurance systems, the methods to measure medical and economic outcomes of AI applications have to follow standardized established procedures. The QALY analysis can be conducted based on different questionnaires to fulfill these requirements, and most studies follow the EQ-5D and the SF-6D format (see Appendix in Supplementary Material) (41).

Still, for existing studies, the quality of the respective impact measurements was often too low to produce reliable and valid results that could serve as basis for a well-founded decision about an AI implementation. This quality can be assessed through the so-called Preferred Reporting Items for Systematic Reviews and Meta-Analyses (PRISMA) and Consolidated Health Economic Evaluation Reporting Standards (CHEERS) (42). The PRISMA guidelines should be used to identify the result report as a systematic review, meta-analysis, or both. The CHEERS criteria support the assessment, as the most common mistakes include items that are not reported in the study. This is of particular relevance as Iribarren et al. outlined that distinct items were missing in up to three-quarters of the publications about the impact of AI applications (43).

The medication selection and dosing company CURATE.AI reported in a cutting edge publication that, based on individually collected data, the adequate drug and respective dosing could be determined with limited side effects. An additional validation of the medical and economic impact of this solution using QALYs-based measurement, could significantly benefit the roll-out process with institutional payors like insurances and healthcare providers, even internationally (44).

Although further approaches such as comparator evaluation, multi-stakeholder analysis or organizational impact were discussed within prior research, a concrete approach with QALYs and quality criteria is needed immediately in order to generate short term results (45).

### Recommendations for Increased Implementation of AI in the Success Factor Categories

As a starting point, concrete measures have been identified regarding the set-up of the technological infrastructure. First, it shall be tailored to the application segment, differentiating between indication-focused and institutional-focused applications. Second, the data processing structure needs to focus on data access and exchange pathways as well as confidentiality measures. Third, a “privacy-by-design” approach shall be implemented and, the overall technological infrastructure should feature a high degree of adaptability in order to also be able to fulfill changing or upcoming regulatory requirements.

In addition to that, a clear and comprehensive AI policy framework is required. This should distinguish between permissionless and precautionary principles, namely between a risk-allowing “fast response” approach and a more cautious “safety first” approach. Furthermore, it should contain principles for AI decision-making protocolling in terms of the time taken for a decision, the number of scenarios considered, and the accuracy of the result obtained by AI to assess AI decisions ex-post. Finally, it must be possible to attribute accountability, liability, and culpability between the involved stakeholders, both human and AI, within the framework.

Furthermore, methodologies and metrics for assessing the medical and economic impact of AI applications must be refined and medical and economic impact assessments have to be intensified significantly. Such assessments should rely on cost-utility estimates and, in particular, on QALYs. Furthermore, we believe that it is indispensable that standardized quality criteria such as the CHEERS and PRISMA criteria (e.g., using a EuroQol-5D questionnaire) are applied so that the results can be evaluated not only by physicians, but also by institutional players.

**Figure 7:**
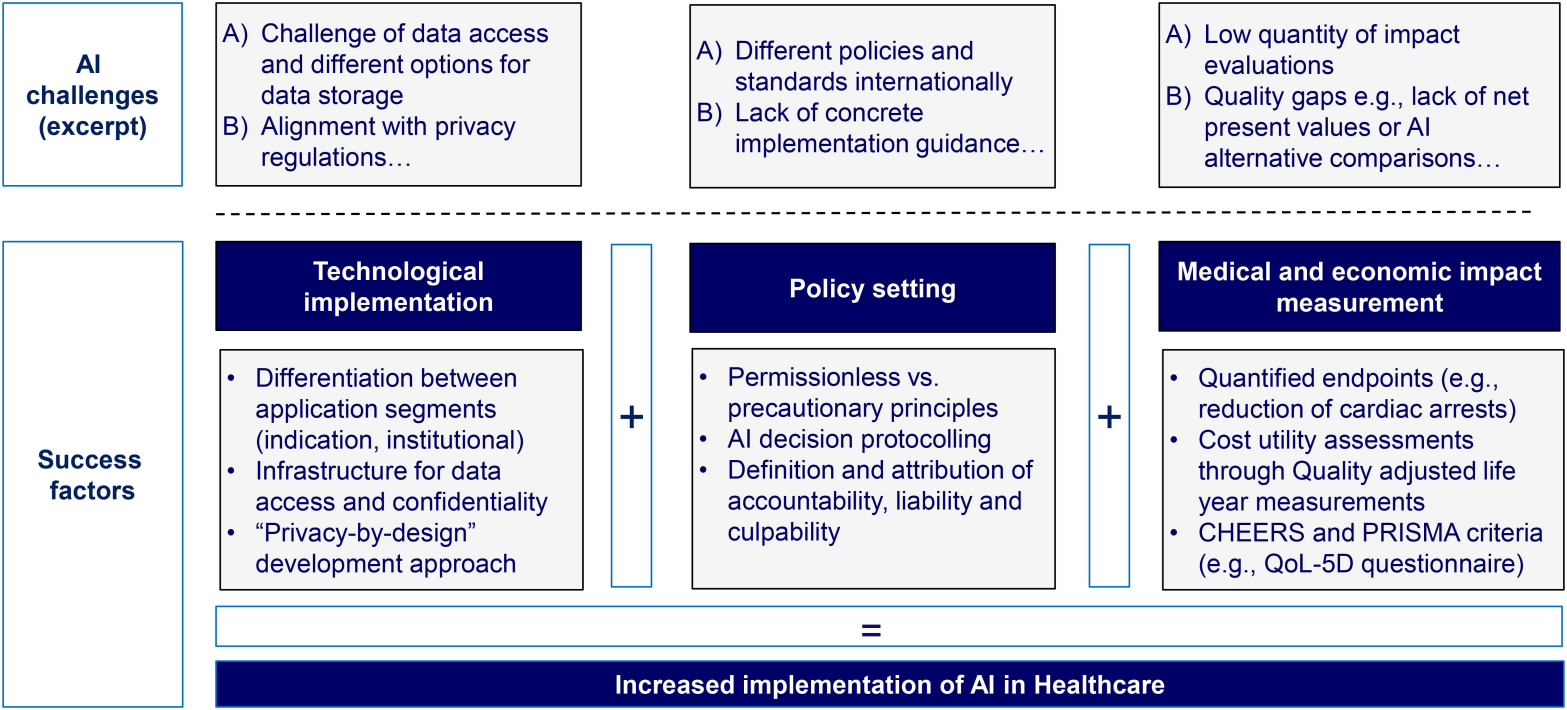
Overview of success factors facilitating the implementation of AI in healthcare (authors).

An overview of the policy, technology, and impact measurement success factors is shown in Figure 7.

## Discussion

We systematically identified success factors that significantly facilitate the implementation of AI in healthcare based on existing academic research and real-world AI applications. In the following, we highlight some limitations.

First, an analysis of additional real-life AI application cases would have provided further relevant insights for the analysis. However, there is no open-access information or there are confidentiality clauses about technological features and economic impact independently of the databases used. Second, academic publications sometimes provide research results with a significant time delay due to the elaborated research process, such as data collection and analysis. Thus, research on very recent developments such as AI policy frameworks, frequently has not yet been conducted or published. Third, there are significant differences across categories. For example, an AI-supported medication adherence system and an AI-driven robotic surgery software are subject to different policy, technological and medical as well-economic impact measurement requirements. As a consequence, success factors will have to be weighted according to the Digital Health and AI conditions in each healthcare system.

Due to these limitations, several further success factors could not be included in the model, but should be a focus of further research and are here briefly discussed.

First, it is important to build trust and confidence among health professionals and patients. This can be seen, for example, in the discussions on COVID-19 tracking solutions. There are different approaches, e.g., for centralized or decentralized data storage, and in many countries intense political debates took place on data storage and tracking. Therefore, trust-building through open communication with easy to understand and well-presented lines of argument is required, and this would also positively influence the acceptance of physicians as “gatekeepers” for AI.

Second, although the categories for “learned from,” “training,” “testing,” or “validation” data are clearly defined in machine learning, in reality often processes are substantially changed or shortened e.g., no model validation takes place with independent datasets. This significantly affects the underlying specificity and sensitivity of AI solutions. Consequently, a clear set of recommended actions for each category would simplify the planning, programming and review processes. Furthermore, continuous reporting also facilitates ex-post verification processes due to the continuous AI learning process.

Third, the different levels of risk associated with AI need to be more clearly differentiated and for instance, the existing medical product classes in Europe could be tailored to AI solutions. Accordingly, AI solutions associated with higher risk will face more stringent regulation. Similarly, more stringent regulations will also be associated with higher costs for registration, documentation, and regulatory compliance. Thus, the market size must be reasonably large, and common market standards for AI risk levels should be established across all states in the US or all EU countries to provide still convincing arguments for AI development.

In summary, there are various barriers to AI implementation, which are likely to significantly have contributed to the considerable gap between the comprehensive and promising academic research on the high potential of artificial intelligence and the comparably low level of its actual practical implementation. Nevertheless, AI has already been applied in different healthcare sectors and is likely to have a meaningful impact on the entire healthcare industry. In particular, due to intense and steadily growing technological developments, current political developments, as well as the fast-evolving industry landscape, we expect a significant AI-driven transformation of healthcare delivery in the future.

The success factors identified in this paper (1) risk adjusted policy frame with clear accountability, liability, and culpability, (2) application scenario specific data processing structures on the basis of legally compliant and still practical privacy by design infrastructures, (3) comprehensive quantification of the medical and economic impact of AI on the basis of QALYs) can significantly facilitate the implementation of AI in routine healthcare processes. While some of the success factors require input from public institutions, private companies can use the success factor analysis already today to build and scale AI services e.g., through high-quality economic measurements and comprehensive technological planning regarding data processing and privacy-by-design structures. However, the current and upcoming success factors should not be perceived as stand-alone measures. Instead, they are strongly interlinked, and their effectiveness is, thus, interdependent to a certain extent. As such, future research needs to elaborate further on the interaction between optimal policy as well as technological, medical, and economic frameworks.

## Data Availability Statement

The raw data supporting the conclusions of this article will be made available by the authors, without undue reservation.

## Author Contributions

All authors listed have made a substantial, direct and intellectual contribution to the work, and approved it for publication.

## Conflict of Interest

The authors declare that the research was conducted in the absence of any commercial or financial relationships that could be construed as a potential conflict of interest.

## Abbreviations

TUM: Technical University Munich
OPAC: Online public access catalog.
