# Supplementary material for: Success Factors of Artificial Intelligence Implementation in Healthcare
Source: Front Digit Health. 2021 Jun 16;3:594971. doi: 10.3389/fdgth.2021.594971 (PMC8521923; doi:10.3389/fdgth.2021.594971)
Supplement: Supplementary file 1 [file Data_Sheet_1.docx]

**Supplementary Material**


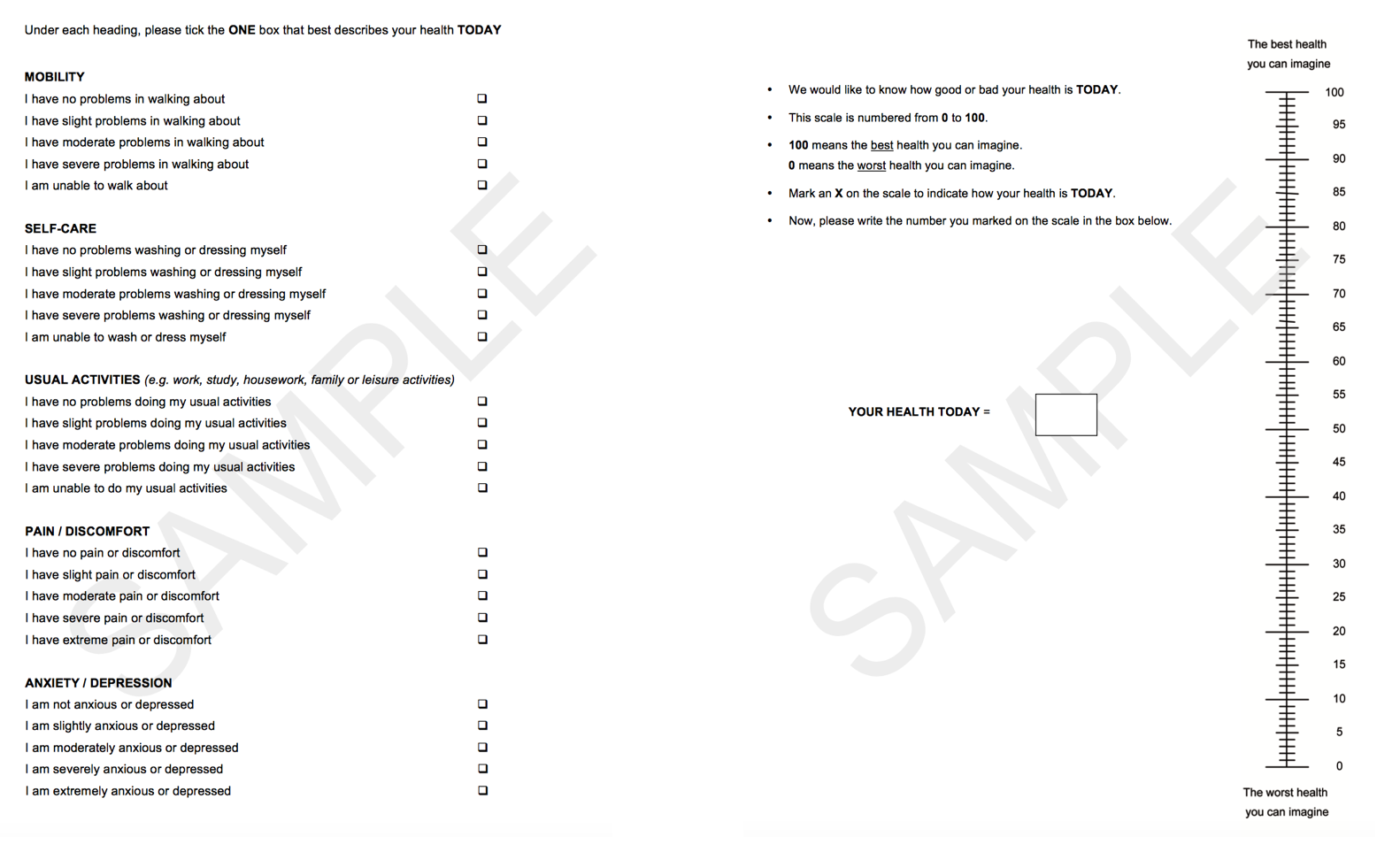


Fig.: An EQ-5D-5L questionnaire of a UK sample version^4^[^7^](https://paperpile.com/c/VTZ6Do/O4Kca)


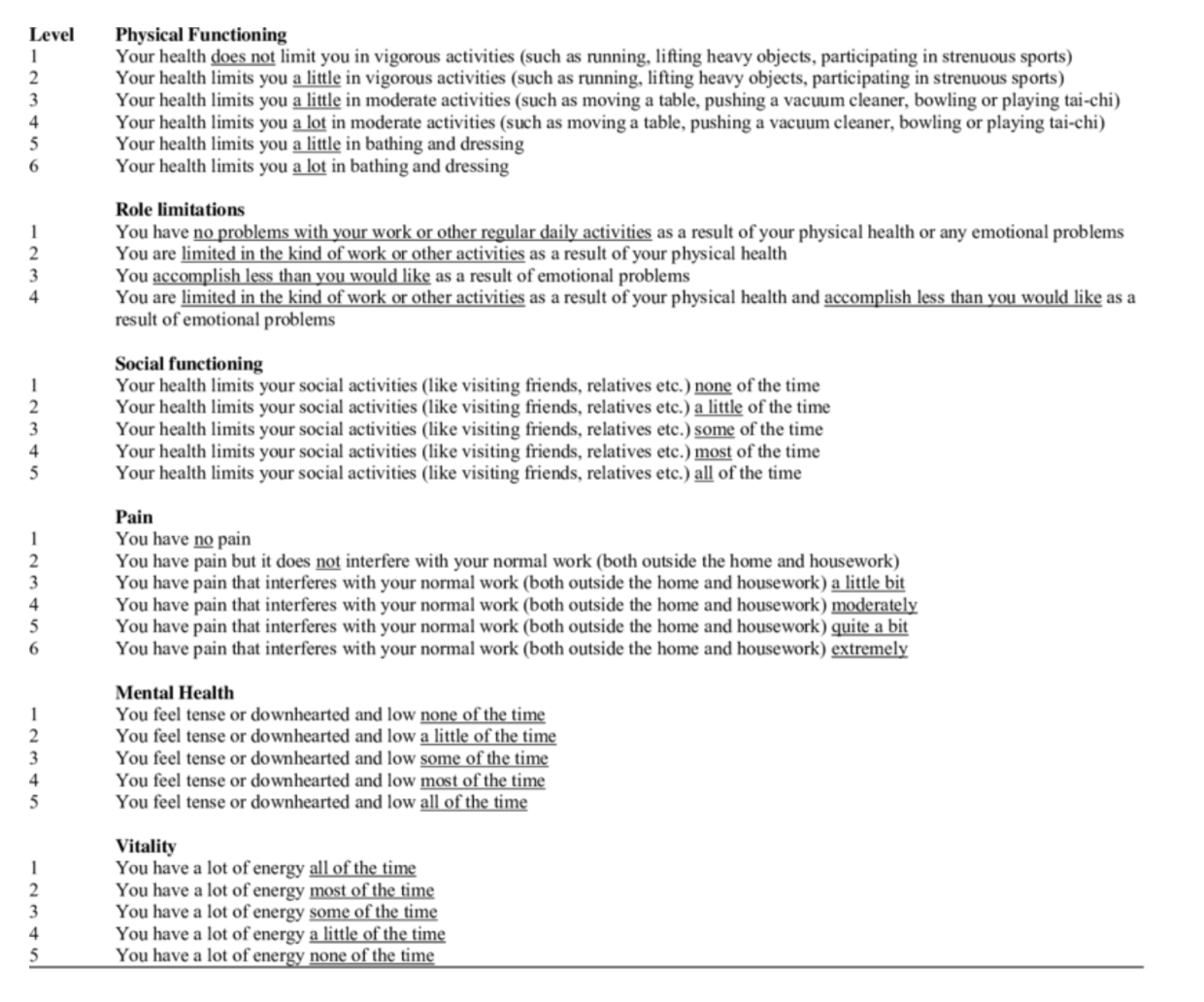


Fig.: A SF-6D questionnaire of a Hong Kong sample version^48^


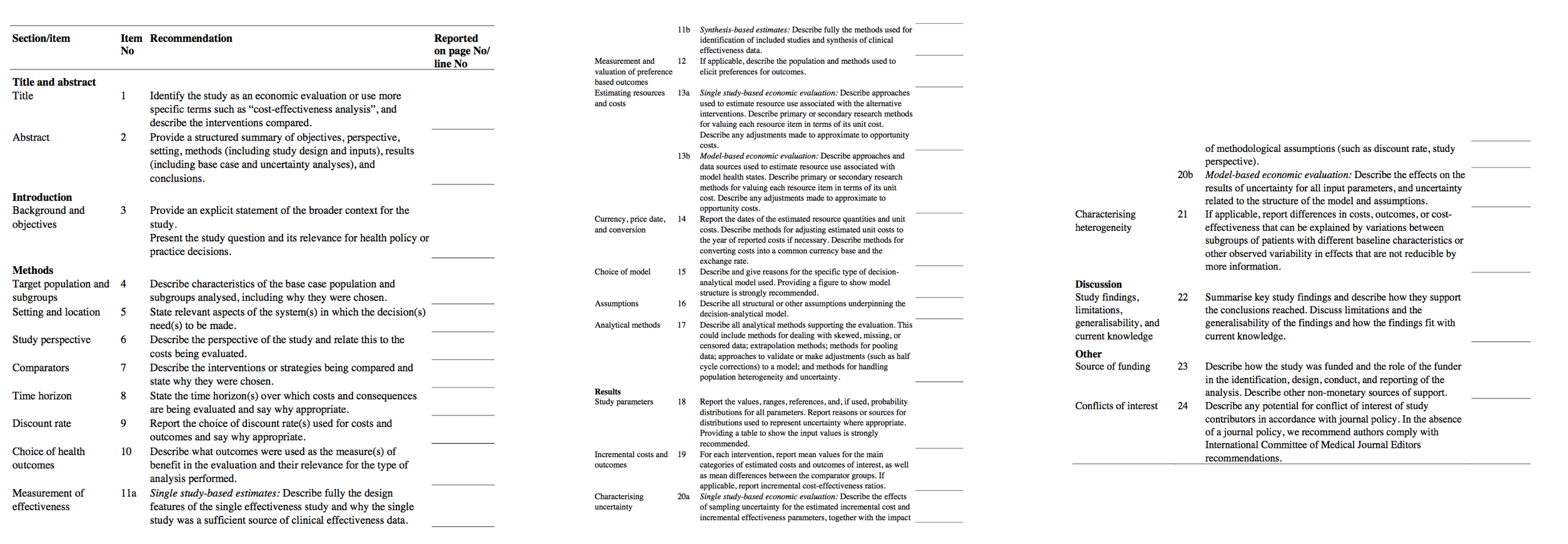


Fig.: CHEERS criteria^15^


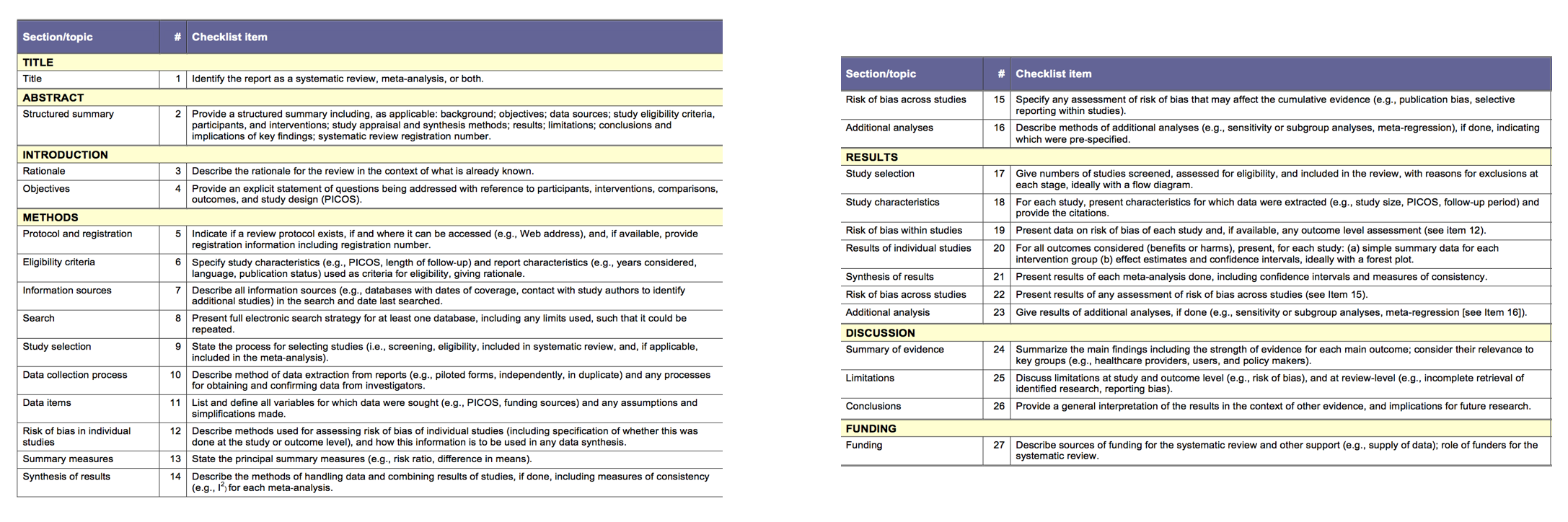


Fig.: PRISMA checklist^49^

**References**

15. Wolff, J., Pauling, J., Keck, A. & Baumbach, J. The Economic Impact of Artificial Intelligence in Health Care: Systematic Review. J. Med. Internet Res. 22, e16866 (2020).

47. Website. See https://euroqol.org/wp-content/uploads/2016/09/EQ-5D-5L_UserGuide_2015.pdf for further information (last access: 18 July 2019).

48. McGhee, S. M. et al. Quality-adjusted life years: population-specific measurement of the quality component. Hong Kong Med. J. 17 Suppl 6, 17–21 (2011).

49. Website. See http://prisma-statement.org/documents/PRISMA%202009%20checklist.pdf for further information (last access: 8 September 2019).
